# Editorial Notes

**Published:** 1924-01

**Authors:** 


### Editorial Notes.

**Sir Leonard Rogers on Leprosy.**

We felt honoured by the visit of Sir Leonard Rogers, C.I.E., to deliver an address at the University of Bristol on Leprosy, a subject on which he has worked so strenuously for many years and towards the eventual suppression of which he has so largely contributed. His researches and laborious investigations, carried out over long periods amongst lepers as well as in the laboratory, seem to constitute a life's work. Yet were all this left undone, his work on cholera, kala-azar, tropical fevers and diseases of the bowels would have made his name honoured in every medical community.

We regret that we cannot publish his lecture entire, but feel sure that our confrères will welcome the opportunity of reading the abstract in this issue.

**Bristol "Milk Week."**

While this issue was going to press the "National Publicity Council," whose Chairman is Mr. Wilfred Bulckley, Milk Controller in the Great War, held its first "milk week" in Bristol.

By reason of the fact that the Bristol Health Committee, under the chairmanship of Mr. Maggs, and with the collaboration of our Medical Officer of Health, Dr. D. S. Davies, had already constituted a "Milk Advisory Committee," comprising representatives of the Health Committee, of farmers and of dairymen, it seems natural that this great movement should be initiated in our city, which has led the way in many modern hygienic developments.

Bristol, through its working committee, has been safeguarding the interests of the citizens and developing the sources of supply, and using its powers to ensure the good quality and freedom from contamination of the milk supply here. This Committee deserves the credit due to its organised effort, which in the course of the "National Publicity Council's Campaign" will doubtless be imitated in many directions.

Valuable contributions at the public meeting held on January 30th were made by Dr. D. S. Davies, who, *inter alia,* mentioned that our national imports comprised 20,000 cwts. of condensed milk weekly, and the total dairy import cost £32,000,000 annually, while much of our home supplied fresh milk was twelve to twenty-four hours old before it reached our population. In the course of their papers Dr. Saleeby stated that both America and Denmark were in advance of Great Britain in their appreciation of fresh milk. Dr. R. A. Askins, our chief school medical officer, urged the need of fresh milk as a preventive of rickets, and showed that condensed milk was inadequate as food in childhood. Dr. J. Middleton Martin, County Medical Officer, emphasised the importance of milk to the growing child, and Dr. J. C. Heaven the value of milk in adequate quantity and good quality for nursing mothers and babies, and said that the Bristol scheme of milk distribution had resulted in a supply of 29,379 gallons of milk, and was one cause of the lessened infant mortality that had taken place.

